# Acute bacterial prostatitis: heterogeneity in diagnostic criteria and management. Retrospective multicentric analysis of 371 patients diagnosed with acute prostatitis

**DOI:** 10.1186/1471-2334-8-12

**Published:** 2008-01-30

**Authors:** Manuel Etienne, Pascal Chavanet, Louis Sibert, Frédéric Michel, Hervé Levesque, Bernard Lorcerie, Jean Doucet, Pierre Pfitzenmeyer, François Caron

**Affiliations:** 1 Infectious and Tropical Diseases department, and Groupe de Recherche sur les Antimicrobiens (GRAM-EA2656), Rouen University Hospital, Rouen, F-76031, France; 2 Infectious and Tropical Diseases department, Dijon University Hospital, 21000 Dijon, France; 3 Urology department, Rouen University Hospital, Rouen, F-76031, France; 4 Urology department, Dijon University Hospital, 21000 Dijon, France; 5 Internal Medicine department, Rouen University Hospital, Rouen, F-76031, France; 6 Internal Medicine department, Dijon University Hospital, 21000 Dijon, France; 7 Geriatric department, Rouen University Hospital, Rouen, F-76031, France; 8 Geriatric department, Dijon University Hospital, 21000 Dijon, France

## Abstract

**Background:**

There is currently a lack of consensus for the diagnosis, investigations and treatments of acute bacterial prostatitis (AP).

**Methods:**

The symptoms, investigations and treatments of 371 inpatients diagnosed with AP were analyzed through a retrospective study conducted in four departments – Urology (U), Infectious Diseases (ID), Internal Medicine (IM), Geriatrics (G) – of two French university hospitals.

**Results:**

The cause of admission, symptoms, investigations and treatments depended markedly on the department of admission but not on the hospital. In U, patients commonly presented with a bladder outlet obstruction, they had a large imaging and functional check-up, and received alpha-blockers and anti-inflammatory drugs. In ID, patients were febrile and received longer and more appropriate antibiotic treatments. In G, patients presented with cognitive disorders and commonly had post-void urine volume measurements. In IM, patients presented with a wide range of symptoms, and had very diverse investigations and antibiotic regimen.

Overall, a 3:1 ratio of community-acquired AP (CA-AP) to nosocomial AP (N-AP) was observed. Urine culture isolated mainly *E. coli *(58% of AP, 68% of CA-AP), with venereal agents constituting less than 1%. The probabilistic antibiotic treatments were similar for N-AP and CA-AP (58% bi-therapy; 63% fluoroquinolone-based regimen). For N-AP, these treatments were more likely to be inadequate (42% *vs. *8%, p < 0.001) and had a higher rate of bacteriological failure (48% *vs. *19%, p < 0.001).

Clinical failure at follow-up was more common than bacteriological failure (75% versus 24%, p < 0.001). Patients older than 49 had more underlying urinary tract disorders and a higher rate of clinical failure (30% versus 10%, p < 0.0001).

**Conclusion:**

This study highlights the difficulties encountered on a daily basis by the physicians regarding the diagnosis and management of acute prostatitis.

## Background

There is a current lack of agreement upon guidelines for the diagnosis and treatment of male urinary tract infections (UTI), in particular concerning acute prostatitis (AP). Indeed, the current NIH classification of prostatitis provides a rather vague description of AP clinical symptoms, referred to as "signs of acute UTI" [1]. Some guidelines reached an agreement for the diagnosis of uncomplicated UTI in male, while others considered that "any UTI has a potential for a prostatic involvement" [2,3]. In AP, some authors recommend to treat initially for *Neisseria gonorrhoaea *and *Chlamydiae trachomatis *in young adults [4]. Conversely, others recommend a treatment only for *Enterobacteriacae *[5]. The antibiotic treatment duration varies from 10 days to 6 weeks [4,6]. This situation is different from UTI in women, where the guidelines for the diagnosis and treatment are very similar in different countries [4,2,7,8,6].

There is a need to analyze how clinicians from different departments and backgrounds define AP before attempting to propose a consensus for AP management. Unlike previously published studies that focused on highly selected patients in urological settings or in prostatitis centers, this study presents the analysis of a large group of patients treated for AP in eight different departments of two French university hospitals [9-11].

## Methods

### Study design

This study presents a retrospective analysis of AP diagnosis and global management. It was conducted in four departments (Infectious Diseases, Urology, Internal Medicine, Geriatrics) of two French university hospitals for a total of eight departments. In these departments, no institutional guidelines for the management of AP were available.

### Patients

Patients with a final physician-assigned diagnosis of AP were included in the study. They were admitted between January 1st 1998 and December 31st 2003 to the above listed departments, with the restrictions described below. Patients preliminarily diagnosed with AP were selected from the central database of each hospital. Patients with uncompleted or missing chart were not included, neither those where the final diagnosis was reported as being different from the preliminary diagnosis (i.e., mistake in data capture).

### Data collection and analysis

All the data was collected from the medical charts by the same investigator (M.E.) using a computerized standard form. The data stated and prescribed in the file by the physicians, the biological data, and the interpretation of the specialist for any complementary investigation (*i.e. *radiologist report for CT-scan) were recorded and analyzed. The data included age, medical history, mode of acquisition, cause for admission, clinical symptoms, biological and imaging investigations, antibiotic treatments, and evolution after discharge. The number of co-morbidities was documented; the most common being progressive neoplasia, HIV infection, immunosuppressive therapy, unstable diabetes mellitus, chronic alcoholism, and neurological disorders affecting the micturition such as paraplegia, dementia or a bedridden state. Haematuria at admission was defined as macroscopic bleeding in urine or blood detection in urine with a rapid strip test. For urine analysis, a leukocyte count greater than 10/mm^-3 ^and a bacterial count greater than 10^4 ^CFU/mL were considered to be significant [3]. The antibiotic treatment was considered to be inadequate when the isolated strains were resistant to all the antibiotics prescribed. The criteria used for microbiological and clinical failure were those defined in the IDSA/FDA guidelines for the evaluation of new anti-infective drugs for the treatment of UTI [3]. A microbiological treatment failure was defined as a positive urine culture (greater than 10^3 ^CFU/mL in a symptomatic patient or 10^5 ^CFU/mL in an asymptomatic patient) at the follow-up visit 5–9 days or 4–6 weeks post-treatment. A clinical treatment failure was defined as the presence of urinary symptoms at any time up to and including the final follow-up visit.

### Expression of results and statistical analysis

The results were expressed globally, according to the department, to the community/nosocomial acquisition of the infection, or to the age of the patients. Using guidelines established in the French consensus, nosocomial UTIs included all urinary infections related to health care facilities, either hospital-acquired or occurring among outpatients with a urinary catheter [12]. The statistical analysis was conducted with Statview^® ^5.0 software (SAS Institute), using Khi-2 tests for quantitative data analysis, and student t-test to compare qualitative and quantitative data. To identify prognosis factors, a univariate analysis was conducted using a logistic regression test. Significant data were studied using a multivariate logistic regression analysis. A categoritical analysis regression tree using JMP software^® ^(SAS Institute) was used to determine an age cut-off significantly associated with an increased rate of clinical failure at follow-up. For all statistical analysis, a p value lower than 0.05 was considered to be significant.

## Results

### Patient inclusion

Between 1998 and 2003, 2170 male patients admitted in the 8 above-mentioned departments presented a final UTI diagnosis. Among them, 586 patients (27%) had a final diagnosis of AP. The chart review resulted in the exclusion of 215 patients (130 missing or uncompleted files, 85 erroneous diagnosis codes). A total of 371 patients were included in the study, 231 from Rouen University Hospital and 154 from Dijon University Hospital, accounting for a 6:4 ratio in accordance with the respective size of the Rouen and Dijon population areas. The distribution of the patients, among the 4 departments and between the hospitals, was homogenous from one year to another, with 48% of the patients in Urology, 31% in Infectious Diseases, 13% in Internal Medicine and 8% in Geriatrics.

### Clinical history and symptoms

**Figure 1:**
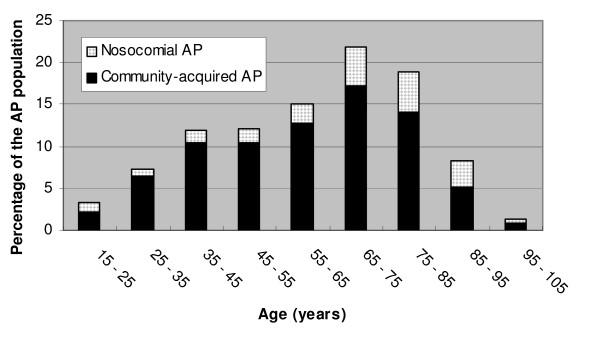
Distribution of 371 patients with acute prostatitis (AP) according to their age and to the mode of contamination.

The mode of contamination and the medical history versus the department are presented in Table 1. Regardless of the department (and the hospital, data not shown), a 3/1 ratio was observed between community-acquired (CA-AP) and nosocomial (N-AP) acute prostatitis. In contrast, the medical history varied between the departments in regards to age, the number of co-morbidities and the urological background. As presented in figure 1, 50% of the patients were over 65 years old whereas 10% were under 35. A past history of UTI was noted in 37% of the patients who were treated with antibiotics on an average duration of 9 days, and a prostatic involvement was specifically noted in 40% of these patients. The following trends in the causes of admission and the clinical symptoms during the course of the disease were noted throughout the different departments: urinary symptoms in Urology, fever and chills in Infectious Diseases, cognitive disorders in Geriatrics, and variable symptoms such as malaise or weight loss in Internal Medicine. They are presented in Table 2.

**Table 1:** Mode of contamination and medical history of 371 patients with acute prostatitis

	**Total patients**	**Department of admission**			
		
		**Urology**	**Infectious Diseases**	**Internal Medicine**	**Geriatrics**
	n = 371	N = 178	n = 115	n = 48	n = 30
**Mode of contamination**					
Community-acquired	293 (79%)	140 (79%)	91 (79%)	41 (86%)	21 (71%)
Nosocomial	78 (21%)	37 (21%)	24 (21%)	7 (14%)	9 (29%)
Hospital acquisition	58 (75%)	26 (69%)	18 (75%)	5 (72%)	8 (87%)
Outpatient with urinary catheter	20 (25%)	11(31%)	6 (25%)	2 (28%)	1 (13%)
**Medical history**					
Age (years)					
Median	61	57	60	66	84
Range	18–99	19–96	18–88	22–99	69–96
Number of co-morbidities					
≤ 1	345 (93%)	175 (98%)	113 (98%)	33 (69%)	23 (78%)
≥ 2	26 (7%)	3 (2%)	2 (2%)	15 (31%)	7 (22%)
Urological background					
Past history of UTI	137 (37%)	53 (30%)	58 (50%)	21 (43%)	6 (19%)
Urinary drainage before admission	42 (11%)	23 (13%)	12 (10%)	4 (8%)	2 (7%)
Past history of anatomical urological disorder	45 (12%)	25 (14%)	14 (12%)	4 (8%)	7 (23%)
Past history of neurological bladder	60 (16%)	18 (10%)	15 (13%)	14 (30%)	12 (40%)

**Table 2:** Causes of admission and clinical symptoms during the course of the disease of 371 patients with acute prostatitis (AP).

	**Total patients**	**Department of admission**			
		
		**Urology**	**Infectious Diseases**	**Internal Medicine**	**Geriatrics**
	n = 371	n = 178	n = 115	n = 48	n = 30
**Cause of admission**					
Fever	297 (80%)	142 (80%)	97 (84%)	37 (78%)	19 (63%)
Urinary symptoms					
Functional symptoms	266 (72%)	153 (86%)	69 (60%)	27 (57%)	15 (50%)
Bladder outlet obstruction	61 (23%)	52 (29%)	9 (8%)	12 (25%)	6 (20%)
Cognitive disorder	14 (4%)	0 (0%)	5 (4%)	4 (8%)	10((33%)
Miscellaneous symptoms	28 (8%)	0 (0%)	2 (2%)	21 (44%)	7 (23%)
**Main clinical symptoms during the course of AP**					
Fever	297 (80%)	154 (84%)	86 (80%)	38 (78%)	19 (63%)
Chills	135 (35%)	47 (25%)	60 (56%)	14 (28%)	7 (23%)
Urinary symptoms	266 (72%)	158 (86%)	65 (60%)	28 (57%)	15 (50%)
- burning micturition	143 (54%)	79 (50%)	36 (55%)	23 (82%)	5 (33%)
- pollakiuria	200 (52%)	77 (49%)	35 (54%)	18 (64%)	9 (60%)
- dysuria	79 (30%)	53 (34%)	15 (23%)	7 (25%)	4 (27%)
- bladder outlet obstruction	61 (23%)	46 (29%)	5 (8%)	7 (25%)	3 (20%)
- macroscopic haematuria	46 (17%)	33 (21%)	9 (14%)	2 (7%)	2 (13%)
Pelvic pain	144 (43%)	98 (58%)	27 (28%)	18 (42%)	1 (4%)
Abnormal digital rectal examination	235 (83%)	135 (89%)	55 (70%)	25 (83%)	20 (91%)
- painful prostate palpation	175 (63%)	115 (77%)	31 (39%)	15 (50%)	14 (64%)
- prostatic hypertrophy	152 (54%)	86 (57%)	36 (46%)	16 (53%)	14 (63%)
- prostate irregularity	66 (24%)	44 (30%)	10 (13%)	4 (13%)	8 (22%)

### Microbiological investigations, PSA and CRP evaluation

Urine analysis was performed for 347 (94%) patients. Among the 122 samples that generated sterile urine cultures (35%), 55 (45%) were taken during antibiotic treatment, and 83 (68%) showed a significant leukocyte count. Among the 225 samples that generated positive urine culture (65%), 19 (8%) were taken during antibiotic treatment, and all but one showed a significant leukocyte count. Table 3 displays the bacteriological results of urine cultures versus the mode of contamination. *Escherichia coli *was the main pathogen, representing 58% of the isolated bacteria. Only 26% of the isolated strains in the N-AP cases were *E. coli*. Antibiotic-resistant strains, either naturally (*Pseudomonas aeruginosa*, *Enterococcus sp*) or through acquired mechanisms (quinolone-resistant *E. coli*) were statistically more common for N-AP. A urethral swab was performed for 9 patients and the following results were noted: 3 were sterile, 3 generated the isolation of more than 5 microbial species, 2 generated the isolation of *Chlamydiae trachomatis*, and one generated the isolation of *E. coli*. The Meares and Stamey diagnosis localizing test ("four-glass" test) was performed on 3 patients [13]. In one case, the test was performed despite a previous positive urine analysis (10^3 ^*Enterococcus faecalis*/mL^-1^, and 10^4 ^leukocytes/mL^-1^). In the two remaining cases and despite the absence of leukocytes and bacteria in the urine samples, the patients were diagnosed with AP on the basis of acute urinary symptoms and fever.

**Table 3:** Bacteriological results of urine cultures versus mode of contamination in a series of 371 acute prostatitis (AP)

	**Bacteriological results of urine cultures**			
	
	**Total patients**	**Community-acquired AP**	**Nosocomial AP**	**Community-acquired *versus *nosocomial AP**
	n = 371	n = 295	n = 76	***p *value**
**Urine culture**	347 (94%)	271 (92%)	76 (100%)	0.02
Sterile	122 (35%)	96 (35%)	29 (38%)	0.71
Positive	225 (65%)	178 (66%)	47 (62%)	0.71
One strain	196 (87%)	159 (89%)	37 (79%)	0.09
≥ 2 strains	29 (13%)	19 (11%)	10 (21%)	0.09
**Isolated strains**	270	213 (79%)	57 (21%)	< 0.001
***E. coli***				
All types	157 (58%)	142 (68%)	15 (26%)	< 0.01
Ampicillin-S	95 (61%)	88 (62%)	7 (50%)	0.4
Nalidixic acid-S	119 (76%)	110 (78%)	9 (57%)	0.2
Ofloxacin-S	130 (83%)	120 (85%)	10 (64%)	0.2
Cotrimoxazole-S	122 (78%)	115 (81%)	7 (43%)	< 0.01
***Proteus***	16 (6%)	11 (5%)	5 (9%)	0.5
***KES *group**	24 (9%)	18 (8%)	6 (11%)	0.8
***Enterococcus***	16 (6%)	8 (4%)	8 (14%)	0.02
***P. aeruginosa***	20 (7%)	8 (4%)	12 (21%)	< 0.01
***S. aureus***	8 (3%)	3 (1%)	5 (9%)	0.02
***Others***	29 (11%)	23 (11%)	6 (11%)	0.9

Blood cultures were performed for 260 patients (70%) and were positive for 120 of them. For 70 patients (19%), the isolated strain was an *enterobactaeriacae*, the same strain as in the urine culture. For 50 patients, a single blood culture isolated a coagulase-negative *Staphylococcus*, suggesting contamination.

The prostatic specific antigen (PSA) was measured for 44% of the patients, on an average of the 4^th ^day of admission, range [0–12], and revealed an abnormality (> 4 mg/L^-1^) in 60% of the cases (median value 17 mg.L^-1^; range [0–415]). Most patients had an inflammatory syndrome: 70% of the patients had more than 10,000 WBC (median of 12.7 G/L^-1^, range [800–38100]), 95% of the patients had an erythrocyte sedimentation rate at the first hour exceeding 10 mm (median 60 mm, range [4–120]), and 96% presented a C Reactive Protein (CRP) exceeding 5 mg/L^-1^(median 123 mg/L^-1^; range [<5–445]).

### Imaging and functional investigations

As presented in Table 4, the percentage of patients undergoing imaging or functional investigations of the prostate displayed a marked variation depending on the department. Out of the 371 included patients, 285 (77%) underwent a pelvic or prostatic ultrasound (US) investigation during their hospital stay (218 supra-pubic and 67 endo-rectal US). The prostatic US was considered abnormal for 228 patients (81%) with the following descriptions: prostatic hypertrophy (n = 127), prostatic calcifications (n = 69), ureteral dilation (n = 21), incomplete micturition (n = 20), prostatic tumor (n = 15), or prostatic abscess (n = 10). Incomplete micturition was specifically investigated by post-voiding pelvic US for 60 patients (21%), and was considered abnormal for 36 (60%). Other imaging investigations performed included: abdomen X-ray (n = 48, 13%), intravenous urography (n = 48, 13%), retrograde cystography (n = 22, 6%), uro CT-scan (n = 11, 3%), cystoscopy (n = 7, 2%). An uroflow measurement was performed after discharge for 122 patients (33%) and was abnormal in 61 (50%) of them. Overall, 182 patients underwent a post-voiding residual urine or an uroflow measurement, and an abnormality was detected in 97 of them (53%).

**Table 4:** Percentage of patients undergoing biological, imaging and functional investigations versus admission department

	**Total patients**	**Department of admission**			
		
		**Urology**	**Infectious Diseases**	**Internal Medicine**	**Geriatrics**
	**n = 371**	**n = 178**	**n = 115**	**n = 48**	**n = 30**
**PSA dosage**	163 (44%)	69 (39%)	52 (45%)	27 (55%)	15 (50%)
**Pelvic ultrasound**	286 (77%)	128 (72%)	105 (91%)	32 (67%)	22 (73%)
**Post-void urine measurement**	78 (21%)	37 (21%)	16 (14%)	7 (15%)	18 (59%)
**Uroflow measure**	122 (33%)	77 (43%)	9 (8%)	0 (0%)	0 (0%)
Other investigations*	152 (41%)	85 (48%)	15 (13%)	3 (6%)	3 (11%)

*Other investigations: abdomen X-ray, intravenous urography, retrograde cystography, uro-CT-scan, and cystoscopy.

### Treatment

Table 5 presents the antibiotic treatments, their adequacy as determined by the results of urine culture, and the rates of microbiological and clinical failures versus the acquisition mode and the department. Half of the patients were initially treated with a combination of antibiotics, regardless of the contamination mode of AP. More than 80% of the combinations included aminoglycosides; 56% of the combinations included a 3^rd ^generation cephalosporin and an aminoglycoside. 225 patients (65%) had positive urine cultures for which the treating physician adapted the antibiotic treatment in 59 cases (16%) based on strain susceptibility. The empirical and adapted antibiotic treatments were inadequate for 16% and 7%, respectively, of the total patients. The proportion of inadequate antibiotic treatments showed marked variations depending on the contamination mode of the AP and the department. The median duration of the treatment was 34 days, significantly longer in Infectious Diseases than in Urology (49 days versus 22 days).

**Table 5:** Antibiotic treatment and rates of microbiological and clinical failure versus mode of contamination and department in a series of 371 acute prostatitis (AP).

	**371 AP patients**							
	
	**Total patients**	**Community acquired AP**	**Nosocomial AP**	Community acquired Versus nosocomial AP	**Urology**	**Infectious Diseases**	**Internal Medicine**	**Geriatrics**
	n = 371	n = 293	n = 78	p value	n = 178	n = 115	n = 48	n = 30
**Antibiotic treatment**								
**Empirical choice**								
Bi-therapy	215 (58%)	172 (59%)	43 (55%)	0.7	123 (69%)	63 (55%)	20 (42%)	9 (30%)
Use of fluoroquinolone	234 (63%)	187 (64%)	47 (60%)	0.7	148 (83%)	47 (41%)	20 (42%)	19 (63%)
Use of 3^rd ^generation cephalosporin	113 (30%)	85 (29%)	28 (36%)	0.3	25 (14%)	59 (51%)	22 (46%)	7 (23%)
Use of amino glycosides	195 (52%)	165 (56%)	30 (38%)	0.007	120 (67%)	60 (52%)	14 (29%)	1 (3%)
Use of other classes	44 (12%)	28 (10%)	16 (21%)	0.01	8 (4%)	12 (10%)	12 (25%)	12 (40%)
Inadequate*	42/269 (16%)	17/210 (8%)	25/59 (42%)	<0.001	27/137 (20%)	4/76 (5%)	6/31 (19%)	5/25 (25%)
**Adapted choice**								
Bi-therapy	15 (4%)	13 (4%)	2(3%)	0.7	3 (2%)	11 (10%)	0 (0%)	1 (3%)
Use of fluoroquinolone	285 (77%)	242 (82%)	43 (55%)	<0.001	148 (83%)	85 (74%)	31 (65%)	21 (70%)
Use of 3^rd ^generation cephalosporin	18 (5%)	11 (4%)	7 (9%)	0.1	9 (5%)	1 (1%)	5 (10%)	3 (10%)
Use of cotrimoxazole	52 (14%)	44 (15%)	8 (10%)	<0.001	13 (7%)	33 (29%)	5 (10%)	1 (3%)
Use of other classes	31 (8%)	9 (3%)	22 (28%)	<0.001	11 (6%)	7 (6%)	7 (15%)	6 (20%)
Inadequate*	18/269 (7%)	11/210 (5%)	7/59 (12%)	0.1	14/137 (10%)	1/76 (1%)	1/31 (3%)	2/25 (8%)
**Total duration (days)**	32	34	29	0.13	22	49	33	33
**Bacterial failure at follow-up**	37/153 (24%)	23/124 (19%)	14/29 (48%)	0.002	16/76 (21%)	2/32 (6%)	1/6 (16%)	4/9 (44%)
- same strain	7	3	4		8	0	0	0
- other strain	30	20	10		8	2	1	4
**Clinical failure at follow-up**	137/183 (75%)	98/135 (73%)	39/48 (83%)	0.3	88/123 (71%)	28/36 (78%)	8/10 (80%)	13/14 (92%)

* The adequacy of the treatment was studied for the 169 patients with a positive urine culture and an antibiotic-resistance pattern of the pathogenic strain.

Alpha-blockers and anti-inflammatory drugs were the most commonly used concomitant medications, in 35 and 20%, respectively. Most of these medications were prescribed in the Urology department. A bladder outlet obstruction was present in 90 patients (25%); 45 patients had urine drainage with a Foley catheter and 45 with a supra pubic catheter, without any significant variation in the proportion of bacteraemia (35% vs. 32%, p = 0.9). Ten abscesses were cured surgically in Urology (9 from patients with CA-AP and 1 from a patient with N-AP).

### Course evolution and follow-up

Apyrexia was obtained on average 2.5 days after the start of the treatment, with no significant variation depending on the department. The median duration of hospital stay was 9 days, and varied significantly according to the mode of acquisition (8 days for CA-AP *vs. *14 days for N-AP) and the department (6 days in Urology, 21 days in Geriatrics). The main early complications were severe sepsis (7% of the patients) and prostatic abscess (4%). Twelve patients (3%) died during their stay, 9 with CA-AP (3%), and 3 with N-AP (4%), however the cause of death was not severe sepsis. The rates of bacterial and clinical failure during follow-up are presented in Table 5. Two hundred and sixty four patients (71%) attended a follow-up consultation after discharge, on an average of 35 days after the start of treatment. The results of the 153 available urine cultures performed at follow-up are presented in Table 5. Positive urine cultures at follow-up were significantly more frequent in patients with N-AP than CA-AP, in patients from Geriatrics than other departments, and in patients with a previous history of UTI (p < 0.01). Information about functional urinary symptoms at follow-up was available for 183 patients (Table 5). 137 (75%) patients described the persistence of urinating pain, discomfort, or both, without any significant difference depending on the department. Eighty-five patients attended a second follow-up consultation, with 80% reporting urinary symptoms. Hospital admission in the Infectious Diseases department was associated with a lower proportion of empirically inadequate antibiotic treatment, a longer total duration of treatment, and a lower rate of bacteriological failure than in the other departments; however, the rate of clinical failure was not significantly different. An increased clinical cure rate was associated with the presence of two symptoms at admission: haematuria or painful digital rectal examination. A decreased bacteriological cure rate was associated with various underlying urological disorders or with other factors such as inadequate empirical antibiotic treatment (Table 6).

**Table 6:** Risk factors for clinical and bacteriological failure

	Clinical failure*		
	
	**Univariate analysis**	**Multivariate analysis**	**Odds ratio**
	**P**	**P**	
**Personal history**			
Age	< 0.001	< 0.001	1.040
history of prostate hyperplasia	0.024	NS**	ND^†^
neurological bladder	0.033	NS**	ND^†^
number of co-morbidities	0.004	NS**	ND^†^
**Symptoms**			
urinary symptoms	0.013	NS**	ND^†^
haematuria	0.005	0.012	0.320
painful digital rectal examination	0.014	0.017	0.340
**Underlying urinary tract pathology**			
discovery of prostate hyperplasia	0.005	NS**	ND^†^
other anatomical or functional pathology	< 0.001	0.013	4.720
**Management**			
Anti inflammatory treatment	0.003	0.003	0.350

	**Bacteriological failure**^††^		
	
	**Univariate analysis**	**Multivariate analysis**	**Odds ratio**
	**P**	**P**	

**Personal history**			
Age	0.001	0.002	1.060
history of urinary catheter	0.001	0.010	4.612
neurological bladder	0.002	NS**	ND^†^
number of co-morbidities	0.044	NS**	ND^†^
**Symptoms**			
pollakiuria	0.006	NS**	ND^†^
Dysuria	0.047	NS**	ND^†^
bladder outlet obstruction	0.004	NS**	ND^†^
**Biology**			
*Pseudomonas aeruginosa *infection	0.006	0.013	7.279
infection with 2 or more strains	0.001	0.008	5.329
**Imaging**			
prostate nodular lesion on ultrasound	0.005	NS**	ND^†^
post void residual urine on ultrasound	0.001	NS**	ND^†^
other anatomical or functional pathology	0.004	NS**	ND^†^
**Management**			
inadequate probabilistic antibiotic treatment	< 0.001	0.002	4.570
urinary drainage with Foley catheter	0.002	NS**	ND^†^
urinary drainage with supra pubic catheter	0.039	NS**	ND^†^

*Clinical failure: patient reporting persistent clinical symptoms at the follow-up visit.

** NS: Not Significant, ^†^ND: Not Determined

^††^Bacteriological failure: positive urine culture at the follow-up visit.

### Impact of age on signs, symptoms, investigations, and outcome

Using a categoritical analysis regression tree, among the 264 patients that had a follow-up visit after discontinuation of the treatment, we have noted that an age above 49 was significantly associated with a higher risk of clinical failure at follow-up (90% versus 60%; p < 0.0001). The signs, symptoms and the results of investigations were analyzed among patients below and above 49. Older patients had significantly more nosocomial infections, more co-morbidities, a history of more frequent UTIs, more urinary retention requiring urine drainage and more underlying urological disorders (higher prostatic volume at DRE and US examination, higher PSA levels, more nodules at DRE, higher rate of abnormal uroflow measures). Older patients also had fewer signs and symptoms (less burning micturition, less painful DRE, and less hematuria). Although underlying urological disorders are significantly more common in older patients, the age was associated with clinical failure at follow-up in univariate and multivariate analysis (Table 6).

## Discussion

This study highlights the difficulties encountered on a daily basis by physicians regarding the diagnosis and management of acute prostatitis.

A wide spectrum of clinical features leads to the diagnosis of AP. Indeed, the accuracy of the physician's diagnosis of acute prostatitis may be questioned: some of the patients may present with a pyelonephritis without prostatitis while others (with urinary symptoms and sterile urine cultures) may have chronic abacterial prostatitis. The main clinical features reported on admission tend to vary between the departments because of the recruitment criteria used in each department (fever in Infectious Diseases, urinary symptoms in Urology etc). The results of this study clearly highlight the variations in the definition of AP among departments and practitioners, and raise the question: what are the key symptoms of AP? There is, surprisingly, no evidence-based answer. The NIH revised classification of prostatitis deals with pathophysiology and biological diagnosis, but the clinical features of AP are briefly described as "signs of acute UTI" [1]. Three recent publications retain fever and urinary symptoms as the diagnostic criteria, while prostatic pain at digital rectal examination occurred in 9 to 100% of patients [14-16].

In regards to biological tests, the NIH revised classification of prostatitis does not include the "four-glass-test" as a criteria for AP diagnosis [1]. Moreover, prostatic massage is not recommended during the early phase of AP, because it is painful for the patient and may lead to bacteraemia and sepsis [17]. In fact, none of the only three "four-glass test" performed in this series contributed to the diagnosis.

No routine clinical, biological or imaging test can currently provide evidence that adequately rules out prostatic involvement in male UTI. While the European guidelines reached a consensus about the diagnosis of "uncomplicated UTI in male," the French guideline and one American guideline consider that "any UTI in male has the potential for a prostatic involvement" [5,2,3]. Two recent imaging studies – one performed with prostatic Indium-labeled leukocyte scintigraphy and one performed with a combination of PSA levels and transrectal US-provided evidence supporting a frequent involvement of the prostate in male UTI [14,18]. Both publications report the presence of an inflammatory reaction within the prostate in 90% of cases, even when digital rectal examination was not painful, or when the physician diagnosed an acute pyelonephritis without prostatitis. These data support previous studies reporting a turbulent urine flow in the prostatic urethra, associated with a reflux into the perpendicular, wide open canals of the peripheral zone of the prostate [19,20]. All these statements underline the need to define detailed and consensual diagnosis criteria of acute (*i.e. *NIH type 1) prostatitis, which would be helpful in AP management as well as in study standardization. However, such a goal cannot be reached through a retrospective study.

The spectrum of microbial etiologies reported in our study is similar to those found in complicated female UTI, both in the case of community-acquired infections (two third of *Enterobacteriacae sp*, 5–10% of *Enterococcus sp *and *P. aeruginosa*) as well as for nosocomial infections (large proportion of resistant bacteria) [21,22]. This pattern has been extensively described in the literature for chronic prostatitis [23-25]. However, in the case of AP, there is large variation in the ratio of pathogens reported in the literature, mainly depending on the detection method: some publications reported up to 57% of *Enterococcus *while others reported up to 40% of *N. gonorrhoeae *[26,27]. The prevalence of venereal agents in our study was low, even in male patients under 35 (2%). An underestimation of their representation due to a lack of specific investigation seems unlikely, considering the high rate of positive standard cultures in this age group; this rate is very similar to the rate observed in other age groups. These results are in accordance with recent studies reporting a low prevalence of venereal micro-organisms, and do not support the recommendations suggesting to treat first for *Neisseiria gonorrhea *and *Chlamydiae trachomatis *in young adults [28,26,30,4].

Finally, a striking 75% rate of clinical failure was noticed in our study. This high value is to be handled carefully, due to the large number of patients not attending follow-up consultation. However, despite the higher incidence of resistant micro-organisms in N-AP than in CA-AP, the empirical antibiotic treatments prescribed in both cases were similar, leading to inadequate antibiotic treatment in 42% of the cases and a higher rate of bacteriological failure. Moreover, the clinical failure rate being much higher than the bacteriological failure rate suggests that antibiotic treatment frequently induces an incomplete resolution of the symptoms, even without any early infection relapse, possibly because of a persistent underlying urological disorder. This finding is supported by a previous urological study of AP, where abnormalities requiring surgical correction were detected in 24% of the patients after a check-up including digital rectal examination, prostatic ultrasound, post-void residual urine measurement and uroflow measurement [18]. In our study, even though uroflow or post-void residual urine measurement were performed in only 33% of the patients, half of them were abnormal. Patients older than 49 had both higher rates of clinical failure and higher rates of underlying urological disorders, and thus might be the target population for prostate-centered investigations. Surprisingly, in our study, initial haematuria, and painful digital rectal examination at admission were associated with a better clinical outcome and were more common in younger patients. Our hypothesis is that the above symptoms are fully related to the infection (*i.e*., not to an underlying abnormality of the prostate), and therefore are easier to control with the appropriate antibiotic treatment.

## Conclusion

In conclusion, this study conducted among inpatients admitted to different hospital units and diagnosed with AP, shows that the clinical features associated with this common infection vary between departments and clinicians. Patients receive different treatments depending on the various departments but overall similar among different hospitals. This heterogeneity in diagnosis criteria and management of AP reflects both the lack of consensual vision in the literature and the difficulties encountered on a daily basis by the physicians.

## Competing interests

The author(s) declare that they have no competing interests.

## Authors' contributions

M. Etienne was the principal investigator and had full access to all of the data in the study and takes responsibility for the integrity of the data and the accuracy of the data analysis.

M. Etienne acquired, interpreted and analyzed all the data, and wrote the manuscript. F.C designed the study, and substantially revised the manuscript. P.C critically revised the manuscript, suggested the search for prognosis factors, helped in statistical analysis and improved the general draft. L.S, H.L, J.D, F.M, B.L, P.P were in charge of the patients, provided full access to data, reviewed carefully and improved the manuscript. All authors read and approved the final manuscript.

## Pre-publication history

The pre-publication history for this paper can be accessed here:


